# A dual-omics approach for profiling plant responses to biostimulant applications under controlled and field conditions

**DOI:** 10.3389/fpls.2022.983772

**Published:** 2022-09-26

**Authors:** Ali Baghdadi, Maria Cristina Della Lucia, Matteo Borella, Giovanni Bertoldo, Samathmika Ravi, Walter Zegada-Lizarazu, Claudia Chiodi, Elena Pagani, Christian Hermans, Piergiorgio Stevanato, Serenella Nardi, Andrea Monti, Francesca Mangione

**Affiliations:** ^1^ Department of Agricultural and Food Sciences, University of Bologna, Bologna, Italy; ^2^ Department of Agronomy, Food, Natural Resources, Animals and Environment, University of Padova, Legnaro, Italy; ^3^ Crop Production and Biostimulation Laboratory, Brussels Bioengineering School, Université libre de Bruxelles, Brussels, Belgium; ^4^ Sipcam Italia S.p.A. belonging together with Sofbey SA to the Sipcam Oxon S.p.A. Group, Pero, MI, Italy

**Keywords:** *Ascophyllum nodosum*, biostimulant, crop yield, plant physiology, tomato, transcriptome

## Abstract

A comprehensive approach using phenomics and global transcriptomics for dissecting plant response to biostimulants is illustrated with tomato (*Solanum lycopersicum* cv. Micro-Tom and Rio Grande) plants cultivated in the laboratory, greenhouse, and open field conditions. Biostimulant treatment based on an *Ascophyllum nodosum* extract (ANE) was applied as a foliar spray with two doses (1 or 2 l ha^-1^) at three different phenological stages (BBCH51, BBCH61, and BBCH65) during the flowering phase. Both ANE doses resulted in greater net photosynthesis rate, stomatal conductance, and fruit yield across all culture conditions. A global transcriptomic analysis of leaves from plants grown in the climate chamber, revealed a greater number of differentially expressed genes (DEGs) with the low ANE dose compared to the greater one. The second and third applications induced broader transcriptome changes compared to the first one, indicating a cumulative treatment effect. The functional enrichment analysis of DEGs highlighted pathways related to stimulus-response and photosynthesis, consistent with the morpho-physiological observations. This study is the first comprehensive dual-omics approach for profiling plant responses to biostimulants across three different culture conditions.

## Introduction

Modern agriculture is seeking eco-friendly ways to sustain crop productivity and reduce the dependency towards chemical fertilizers (Xu and Geelen, 2018). Conventional agricultural practices mainly rely on synthetic agrochemicals. They are uneconomical and harmful to the environment and human health (Dookie et al., 2021). Over the past decades, plant biostimulants have become sustainable inputs for agriculture (De Saeger et al., 2020; Del Buono, 2021). The global market of plant biostimulants reached up to USD 2 billion in 2019, and it is projected to reach USD 3.93 billion, with an average Compound Annual Growth Rate (CAGR) of 11.54% between 2020 and 2025 (previously 10.95% between 2015 and 2020) (Dunham and Trimmer, 2020). In this expansion scenario, the concept of biostimulant activity relates to current and future regulations and regulatory prescriptions regarding the placement of plant biostimulants in the market (Lucini and Miras-Moreno, 2020).

One of the first formally agreed-upon definitions of plant biostimulant was outlined by the EU Fertilizer Regulation 2019/1009. This was a milestone in recognition of the biostimulation concept, that frames these products in a discrete class of fertilizers based mainly on their function. Accordingly, a plant biostimulant is a product stimulating plant nutrition processes independently of the product nutrient content, with the sole aim of improving one or more of the following characteristics of the plant or the plant rhizosphere: i) nutrient use efficiency, ii) tolerance to abiotic stress, iii) quality traits or iv) availability of confined nutrients in the soil or rhizosphere.

Another aspect to consider when evaluating the effects of biostimulants is the method of application. Biostimulants can be applied as a seed treatment, soil preparations -or drenches-, or sprayed on leaves and other aerial organs (Drobek et al., 2019). Different factors should be considered, like the type of substance applied, the expected effects on the plant, the crop species and phenological stage, the growing conditions, and the agricultural practices. Plant nutrient absorption happens both through leaves and roots: seaweed-based extracts can be utilized as root treatments for the soil and/or foliar sprays. Both application methods can be equally effective to improve plant stress tolerance, growth, and yield (Ali et al., 2016). Soil applications can modify the biological and physical soil properties by stimulating soil microflora, improving water retention and nutrient availability (Battacharyya et al., 2015). Nonetheless, the foliar application is more convenient for characterizing biostimulant effects on plant biochemistry and physiology because it directly targets the aerial organs. On the contrary, soil application introduces more complexity due to the buffer effect exerted by the biological, chemical, and physical soil properties.

The physiological characterization of biostimulant function and the science-driven product development have become a prerequisite for introducing effective and reliable plant biostimulants on the market. Nevertheless, most of these products are complex substances or mixtures. Such complexity raises the challenge of understanding the modes of action. Currently, the implementation of phenotyping with omics approaches moves research on plant biostimulants forward to identify key information on plant metabolic pathways and developmental processes (Yakhin et al., 2017; Nardi et al., 2021). Precisely, the integration of omics technologies (i.e. metabolomics, phenomics, transcriptomics) enables a comprehensive molecular and physiological characterization of plant biostimulant effects (Della Lucia et al., 2022; Franzoni et al., 2022). Such technologies are very informative tools, whose potentialities can be maximized by setting an experimental design that considers different degrees of environmental variability to better describe plant biostimulants modes of action. However, the traits associated with the biostimulant action strongly depend on the environmental conditions. Therefore, the characterization of the impact of the product on crops and its technical definition requires the experiments to be carried out in different field conditions and with dedicated multidisciplinary study plans, aimed at dissecting the complexity of the plant response in the open field (Ashour et al., 2021; Della Lucia et al., 2021).

Undeniably, crops grown in the open field are exposed to multiple abiotic stresses and heterogeneous conditions which are hardly reproducible in laboratory conditions. Moreover, the plant phenotype is directly affected by the environment, and observed phenotypic variables reflect these interactions. Accordingly, plant biostimulants screened in a controlled environment can perform differently than in field conditions (Rouphael et al., 2018). Several reasons account for these observed discrepancies. For instance, weather conditions can reduce the biostimulant efficacy after foliar treatment (Pecha et al., 2012). Furthermore, soil chemical and physical properties as well as the native-microbial composition exert specific effects on plants (Fadiji et al., 2022). In practice, plant biostimulants are evaluated first in controlled environment to speed up the selection process of the most interesting products and eventually in the field. However, studies are usually focusing on one or another environment, without gaining a complete functional characterization of plant biostimulants.

Among biostimulants, seaweed-based extracts are widely adopted in cultivated plants. Especially the brown inter-tidal seaweed *Ascophyllum nodosum* is widely used for the formulation of commercial products and it has shown to beneficially influence the plant ability to face biotic and abiotic stresses and to improve plant growth (Shukla et al., 2019). The bioactivity of seaweed extracts is not homogeneous among different products, as it strongly depends upon the extraction method and the harvest season and geographic location. (Carrasco-Gil et al., 2018). The main constituents of seaweed extracts are polysaccharides, fatty acids, amino acids, mineral compounds, phytohormones, and secondary metabolites (phenolic compounds, vitamins, and their precursors) (Pereira et al., 2020). The application of *Ascophyllum nodosum* extract (ANE)-based biostimulants is reported to increase chlorophyll content and yield in tomato and pepper, to improve the yield and quality of the harvested product in grapevine (improved berry size, weight, and firmness) and olive (increased oil content and fatty acids composition), to enhance photosynthetic rates and antioxidant enzymes activities of soybean, and to promote net photosynthetic rate, water and nutrient use efficiency, and sucrose accumulation in sugarcane (Battacharyya et al., 2015; Chen et al., 2021; Chandra and General, 2022).

This study focuses on transcriptomic and physiological responses in tomato plants, after a foliar application of *Ascophyllum nodosum* extract (ANE). Through a dual-omics approach, molecular targets of ANE were identified by RNA-Seq analysis, and the expression level of the most representative genes was confirmed by qPCR. Complementary morpho-physiological experiments were conducted in a climate chamber, greenhouse, and open field conditions to achieve a comprehensive characterization of the ANE biostimulant.

## Materials & methods

### Experimental design and growing conditions

**Figure 1:**
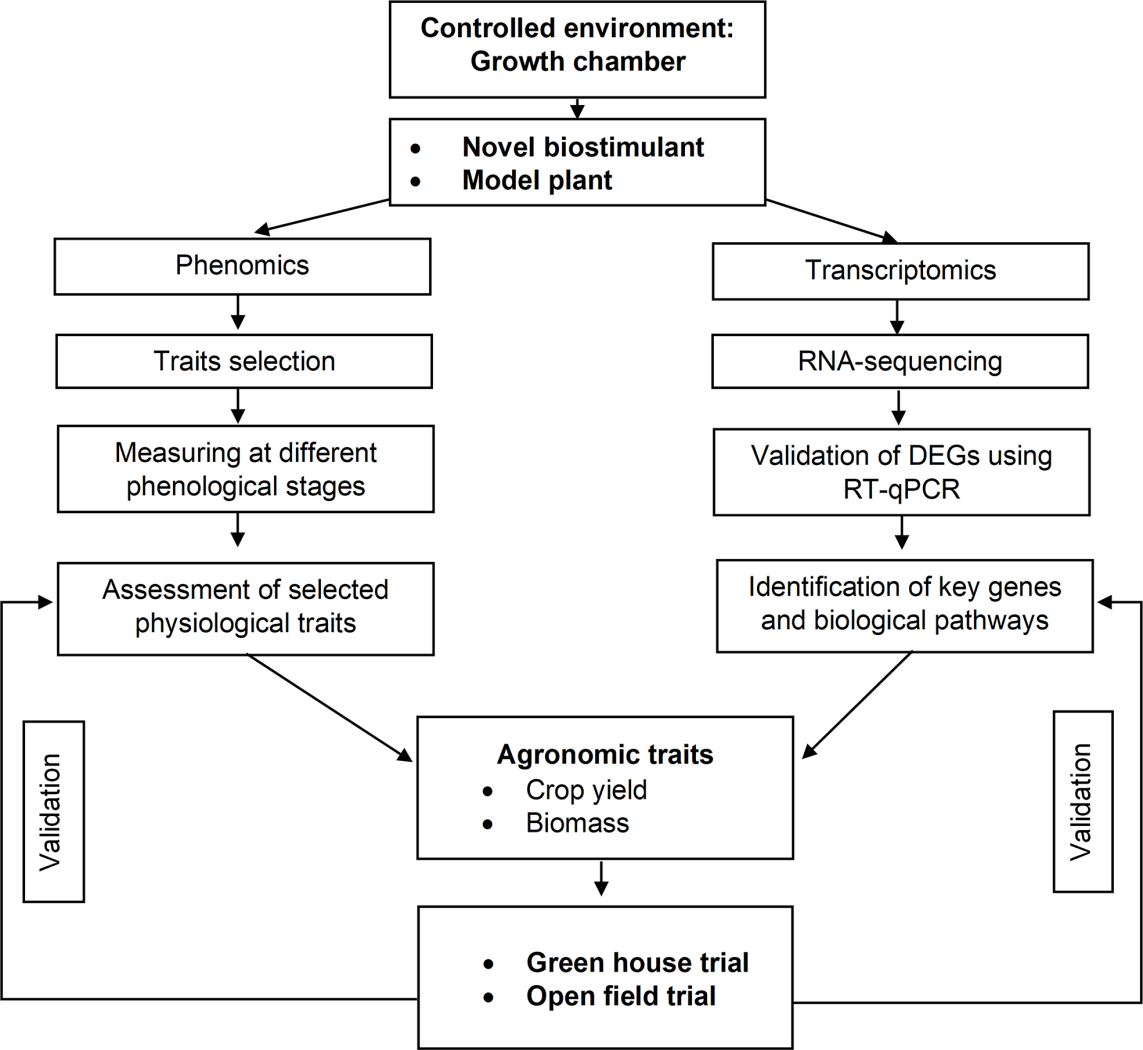
Workflow adopted to assess the effects of a biostimulant in controlled environments and open field. The main steps are briefly described. The first step includes phenomic and transcriptomic analyses conducted in the laboratory and the evaluation of agronomic traits. The last step is the validation of the observed biostimulant effects in the greenhouse and field.

During the years 2020 and 2021, experiments were conducted in three different environments: (i) climate chamber (first year), (ii) greenhouse (second year), and (iii) open field (second year). The adopted workflow is presented in **Figure 1**. The plant material was *Solanum lycopersicum*. The Micro-Tom cv. was grown both in climate chamber and greenhouse, while Rio Grande cv. in the open field.

A biostimulant product based on one extract of the brown alga *Ascophyllum nodosum* (ANE) provided by Sipcam Oxon S.p.A. (Pero, Italy) was applied as a foliar spray. The chemical composition is reported in **Table 1**. We tested different doses of seaweed extract obtained by serial dilutions (from 1:10000 to 1:100) to identify a range of optimal product efficacy in terms of the promotion of plant biomass and fruit yield. These preliminary experiments (data not shown) permitted the selection of two ANE doses: 1 or 2 l ha^-1^. Foliar applications were repeated three times during the reproductive phase at the specific stages: BBCH51 (first inflorescence visible, first bud erected), BBCH61 (first inflorescence: first flower open), and BBCH65 (five or more inflorescences with open flowers) (Meier, 2001).

**Table 1:** *Ascophyllum nodosum* extract (ANE)-based biostimulant composition, provided by Sipcam Oxon S.p.A. .

Characteristics	Values	Unit
Dry matter content	10.9	%
Density	1.046	kg l^-1^
pH (t.q.)	4.6	
Sieve residue at 150 µm-45 µm	0.01 - 0.1	%
Conductivity	4.2	mS cm^-1^
Organic Carbon of biological origin	3.8	%
(% TQ) Mannitol	1.3	%
	13.6	g l^-1^
**Trace Elements**		
Zn	10	ppm
Co	<1	ppm
B	14	ppm
Al	20	ppm
Cu	6.5	ppm
Fe	35.5	ppm
Mo	<0.2	ppm
Mn	4.7	ppm
**Macro-, meso nutrients**		
N tot	0.11	%
P_2_O_5_	0.05	%
K_2_O	0.62	%
Na	0.4	%
Mg (ppm)	895	ppm

### Assay in climate chamber

In the climate chamber, two types of light-emitting diodes (LED) were used: an AE100 and an AE80 at a photon flux density (PFD) of 250-290 and 210-230 μmol photons m^-2^ s^-1^, respectively. The photoperiod was 14h light and 10h darkness. Relative humidity was set to 60% and temperature to 24°C (light)/20°C (darkness). Tomato plants cv. Micro-Tom were cultivated on Klasmann-Deilmann (Germany) substrate: 35% white sod peat 10-25 mm, 45% white peat 0-25 mm, 5% peat fiber, and 15% perlite. The substrate physical and chemical properties are given in **Supplementary Table 1**. Plants with three to four true leaves (30-35 d after sowing) were transplanted in pots with a capacity of 1.2 l. From the third week after sowing, plants were fertilized twice a week with Flortis (Energy blue) NPK (20:20:20). Upon reaching the biostimulant treatment application time, the standard maintenance fertilization was replaced with a formulation entitled to be more suitable for plant development (NPK 15-15-30 Flortis Prod). Each pot was irrigated with 150 ml of water, three times per week. For preparing spray solution, 1.375 g or 2.750 g of ANE were diluted in 1 l of ultra-pure water, respectively corresponding to 1 l ha^-1^ or 2 l ha^-1^ doses. A volume of 10 ml was sprayed on leaves. Control plants were sprayed with an equal volume of ultra-pure water. The trial was arranged as a completely randomized design with seven replicates (pots) each containing one plant.

#### Leaf gas exchange measurements

Leaf gas exchange measurements were done on the youngest fully expanded leaves below the nearest inflorescence, before the first ANE application and 48h after every other application at three phenological phases (BBCH51, BBCH61, BBCH65). Gas-exchange measurements were taken with an infrared gas analyzer (CIRAS 3 PP Systems, Amesbury, MA, USA), under ambient temperature, saturating light of 1,500 μmol photons m^-2^ s^-1^ and 400 μmol CO_2_ surrounding the leaf flux density. The size of the leaf cuvette window was 2.5 cm^2^, and the light was provided by red, green, and blue light-emitting diodes.

#### Yield parameters

In both climate chamber and greenhouse experiments, plants were harvested at the fruit maturity stage. The number of fruits, the fruit weight per plant, and their total biomass were recorded. At harvest, the fresh fruit yield was measured, and the dry weights were recorded after oven-drying the samples at 105°C for 24h.

#### RNA sequencing

Samples treated with two ANE doses were harvested 24h and 48h after treatment for RNA-Seq analysis together with controls.

Two leaf disks were collected around the mid-vein of the distal leaflets of the most recently fully expanded leaf below the nearest inflorescence, from four different plants for each experimental condition. Messenger RNA was directly isolated from frozen and powdered leaf disk pools using the Dynabeads mRNA Direct Micro Kit (Thermo Fisher Scientific, Carlsbad, CA) following the manufacturer’s instruction. The concentration and quality of mRNA were assessed by an Agilent 4150 TapeStation system (Agilent Technologies, USA). Sequencing libraries were prepared from a range of 10-50 ng of poly(A) RNA using Ion Total RNA-Seq Kit v2 (Thermo Fisher Scientific) following the manufacturer’s protocol. The final double-stranded barcoded cDNA libraries were eluted in 15 µl of nuclease-free water. The concentration and size distribution were quantified through D1000 screen Tape (Agilent Tapestation 1500), normalized to get a molar concentration of 100pM, pooled, and sequenced using three Ion 540™ Chips on the Ion Torrent S5 System (Thermo Fisher Scientific).

#### Sequencing data and differential gene expression analysis

Raw reads were filtered to remove the low-quality ones and use reads with a phred-like Q value > 20 for downstream analysis. Bowtie2 (v2.4.2) (Langmead and Salzberg, 2012) was used for mapping the filtered reads to *Solanum lycopersicum* genome (SLv3.0) (NCBI, GenBank accession GCA_000188115.3). The raw transcriptome data obtained are available at the ENA Browser under the name “PRJEB53962 (ERP138777)”. Raw read counts were calculated for all predicted genes using bedtools multiBamCov (Quinlan and Hall, 2010) after processing mapped reads with samtools (v1.11) (Li et al., 2009). To remove less informative data, we filtered out genes with an overall expression level smaller than 20. The DESeq2 R package (v.1.32.0) (Love et al., 2014) was used to perform the inferential analysis and obtain differentially expressed genes (DEGs) across the biological conditions. An adjusted p-value < 0.1 and a log_2_ fold change ≥ |1.0| were set as thresholds of significance to select DEGs. Gene Ontology (GO) enrichment analysis was performed with the web-based toolkit ShinyGO v0.66 (http://bioinformatics.sdstate.edu/go/) (Ge et al., 2020) at an FDR threshold of 0.05, and lollipop plots and tree hierarchical clustering of GO terms were generated on the same online platform.

#### Validation of DEGs using RT-qPCR

Genes differentially expressed across different time points were selected to evaluate their expression levels through RT-qPCR for validation of RNA-Seq results. The validation was performed on biological replicates collected 24h after treatment with 2 l ha^-1^ ANE in the three phenological stages. Primers were designed using the Primer-BLAST tool on NCBI (Ye et al., 2012). The list of primers is shown in **Supplementary Table 2**. A quantity of 3 µg total RNA extracted with a Maxwell^®^ 16 LEV Plant RNA Kit (Promega Corporation, USA) was converted into cDNA using a GoScript Reverse Transcription Mix, Random Primer (Promega Corporation, USA). The RT-qPCR assay was performed using a reaction mix composed of 5 μl of GoTaq qPCR Master Mix (Promega Corporation, USA), 1 μl of cDNA (4 ng μl^-1^), and 0.25 μl of each gene-specific primer in a final volume of 10 μl. Three biological and two technical replicates were performed for each gene. The average Ct values of two internal reference control genes *EFI1* (Solyc06g005060.2; Forward: 5’-CTGTGAGGGACATGAGGCAG-3’, reverse: 5’-CTGCACAGTTCACTTCCCCT-3’) and *UBI* (Solyc07g064130.1; Forward: 5’-GGACGGACGTACTCTAGCTG-3’, reverse: 5’-TCGTCTTACCCGTGAGAGTC-3’) were measured for relative expression analysis using the comparative 2^−ΔΔCt^ method (Schmittgen and Livak, 2008).

### Greenhouse experiment

The greenhouse experiment was carried out in a fully equipped structure with a lighting system (PFD: 300 ± 20 μmol photons m^-2^ s^-1^) adjusted to 14/10h light/dark, 24/20°C light/dark temperature, 60% relative humidity, natural ventilation roof, lateral openings, and horizontal fan systems for air circulation. All the methodological parameters on plant material, growing conditions, treatments, and experimental design were the same as previously described in the climate chamber experiment. After seed germination, Micro-Tom plants with three to four true leaves were transplanted to individual 1.2 l-capacity pots that were arranged in a completely randomized design with seven replications per treatment. Treatments consisted of untreated control and two ANE doses (1 and 2 l ha^-1^) applied as a foliar spray in three phenological stages (at BBCH51, BBCH61, and BBCH65). Leaf gas exchange and yield traits were measured as above described in 2.2.1 and 2.2.2. The percentage of fruit set was computed on six plants (pots) by counting the total number of flowers in the second and third clusters and later, on the same clusters, at full maturity, the number of fruits. The fruit set percentage was calculated as follows:


$$Fruit\;set\;(\%)=\frac{Number\;of\;fruits}{Number\;of\;flowers}\times 100$$

### Field experiment

A field trial was conducted at the experimental farm of the University of Bologna located in Cadriano (Italy) (44° 33’ N, 11° 24’ E) during the growing season of 2021. The cv. Rio Grande was used. Four-week-old seedlings cultivated in a greenhouse on the soil substrate previously described in 2.2 were transplanted to the field. Pre-transplant mineral fertilization consisted of 110 kg ha^-1^ N (slow-release fertilizer), 100 kg ha^-1^ P_2_O_5_, and 200 kg ha^-1^ K_2_O. During the fruit setting plants were enriched with calcium nitrate (foliar, 2 kg 1000 l^-1^). Water was applied by drip irrigation at a rate of 5 l m^-1^ h^-1^ with drippers spaced 40 cm. The first watering was done immediately after transplanting. The amount of water supplied was calculated by both the ETo (reference evapotranspiration (mm day^-1^) climate conditions and by the crop phenological stage expressed by the Kc factor (crop coefficient), using the following formula: crop evapotranspiration or crop water need (ET crop) (mm day^-1^) = ETo × Kc (Brouwer and Heibloem, 1986). Values of the crop factor (Kc) for tomato crop and growth stages were between 0.45-1.15. The monthly and long-term mean (10 years), maximum, and minimum temperature and precipitation during the experimental period are presented in **Supplementary Figure 1**. A composite soil sample was collected before the experiment to determine the physical and chemical characteristics at 0-30 cm depth. The physical and chemical properties of the soil are presented in **Supplementary Table 3**. The experimental set-up was a completely randomized block design with three blocks and four replications per treatment (1 l ha^-1^, 2 l ha^-1^ of ANE, and control). Each plot had a surface of 20 m^2^ (4x5 m) and consisted of four rows. The space between rows was 115 cm and between plants in one row 40 cm. A buffer zone of 3 m spacing was provided between plots. Two ANE doses (1 l ha^-1^ and 2 l ha^-1^) were applied using a hand sprayer three times, specifically at BBCH51, BBCH61, and BBCH65, and were compared with untreated control.

#### Leaf gas exchange, biomass, and fruit yield measurements

Leaf gas exchange measurements were done before the first ANE application (BBCH51) and 48 h after the last one (BBCH65). The measurements were done on the youngest fully expanded leaves below the nearest inflorescence on five plants per treatment in the morning (9.00-11.00 am). The fruit set percentage and fruit fresh and dry weight were measured. To assess the tomato fruit set in the field, the total number of flowers in the second and third clusters were counted in five randomly selected plants within the plot. The fruits were counted at the fruit’s development stage on the same clusters where the total flowers were counted. The fruit set (measured as a percentage) was calculated as a ratio between the fruits and flowers numbers. Fruits harvested at full ripening from 10 plants from the central rows were weighed with an electronic dynamometer. The dry weight of fruits was measured after the samples were oven-dried at 105°C.

### Statistical analysis

The statistical method applied to physiological traits data was the repeated measurements ANOVA model. Productivity traits were subjected to a one-way analysis of variance (p< 0.05), and the differences between samples were determined by the least significant difference (LSD) test. Statistical analyses were carried out using RStudio (version R-4.1.0). Venn diagrams were plotted using ggVenn package from R.

## Results

Physiological and molecular characterization of the ANE-based biostimulant effects were first assessed in laboratory conditions with plants cultivated in a climate chamber and treated at three growth stages. Eventually, plant physiological and yield-related traits were evaluated in greenhouse and open field conditions.

### Effects of ANE treatment on tomato plants grown in culture chamber

#### Leaf gas exchange and yield

**Figure 2:**
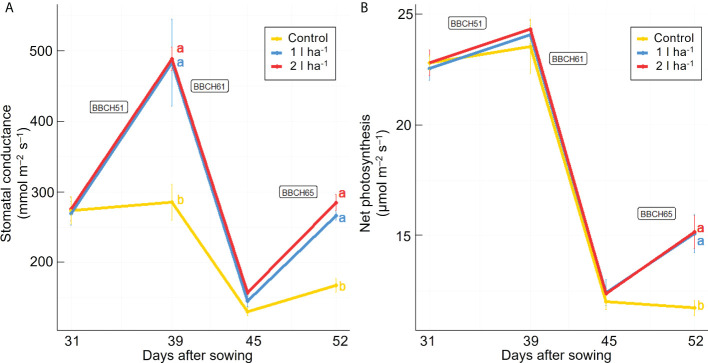
Effect of ANE treatment on photosynthetic parameters in tomato plants cultivated in a climate chamber. Stomatal conductance **(A)** and net photosynthesis **(B)** were measured before the first treatment application and 48h after every ANE leaf application at BBCH51, BBCH61, and BBCH65 in Micro-Tom plants untreated (control) or treated with ANE (1 or 2 l ha^-1^). Each value is the mean of n = 6 observations ± s.e. Different letters indicate a significant difference according to LSD Fisher’s test (P ≤ 0.05).

**Figure 3:**
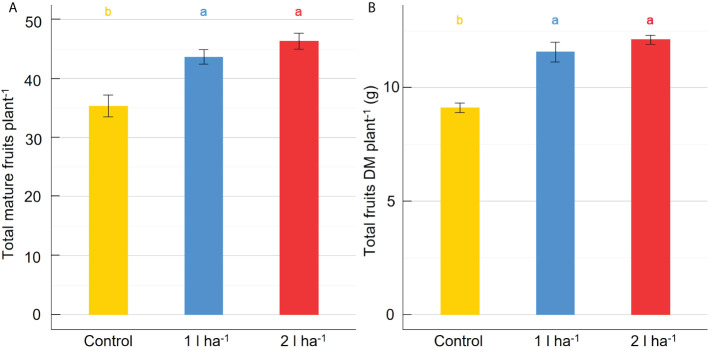
Effect of ANE treatment on fruit production in tomato plants cultivated in a climate chamber. The total number of mature fruits per plant **(A)** and the total fruit dry biomass per plant **(B)** were measured in Micro-Tom plants untreated (control) or treated with ANE (1 or 2 l ha^-1^). Each value is the mean of n = 6 observations ± s.e. Different letters indicate a significant difference according to LSD Fisher’s test (P ≤ 0.05).

Stomatal conductance and net photosynthesis were measured across three different time points after applying the ANE. The average rates were increased significantly (p ≤ 0.05) by the treatment but were not significantly different between the two doses (**Table 2**). The average stomatal conductance was 41% and 36% greater than the control in plants treated with the 2 l ha^-1^ and 1 l ha^-1^ dose, respectively. A significant interaction (p ≤ 0.05) between different doses of ANE and time of application was detected in stomatal conductance and net photosynthesis (**Table 2**). Stomatal conductance was greater in treated plants after the first ANE applications at BBCH51 and BBCH65. A significant effect on net photosynthesis was obtained only after the last ANE application. The difference between the two doses is not significant for both leaf gas exchange parameters (**Figure 2**). At the final harvest, ANE application significantly (p < 0.05) increased the fruit number per plant compared to the control. The plants treated with the two ANE doses showed significantly greater total fruit dry matter than untreated ones. No significant difference was found between the different doses of biostimulant (**Figure 3**).

**Table 2:** Mean values and analysis of variance of photosynthetic parameters after foliar application of ANE (biostimulant, B) at different phenological phases (time, T) in a climate chamber.

Treatment	Stomatal conductance (mmol m^-2^ s^-1^)	Net photosynthesis (µmol m^-2^ s^-1^)
**Biostimulant (B)**		
2 l ha^-1^	302 ± 25.5 a	18.7 ± 1.1 a
1 l ha^-1^	291 ± 29.5 a	18.5 ± 1.1 a
Control	214 ± 16 b	17.5 ± 1.2 b
**Time (T)**		
Before first treatment	273 ± 9.6 B	22.7± 0.3 A
BBCH51	418 ± 31.4 A	23.9 ± 0.4 A
BBCH61	145 ± 4.1 C	12.2 ± 0.2 C
BBCH65	240 ± 13.4 B	14.0 ± 0.5 B
**ANOVA significance**		
B	*	*
T	*	**
B x T	**	*

Data are means ± standard error. Different letters indicate a significant difference according to LSD Fisher’s test (P ≤ 0.05). *, ** significant respectively at 0.05 or 0.01 levels according to ANOVA. BBCH51 (the first inflorescence visible: first bud erects), BBCH61 (first inflorescence: first flower open), BBCH65 (fifth inflorescence).

#### Leaf transcriptome responses

To detect transcriptional changes induced by ANE treatment, mRNA sequencing was conducted on leaves collected 24h and 48h after each application. A total of 252,549,495 single-end reads were generated by the sequencing runs, with an average of 7.015 10^6^ raw reads per sample. The overall alignment rate after mapping to the *S. lycopersicum* reference genome was on average 78.85%.

A principal component (PCA) plot with the log2 normalized read counts (**Supplementary Figure 2**) shows samples are mainly clustered according to the collection phenological stages (**Figure S2A**). However, samples collected at the beginning of the reproductive phase (BBCH51) are not tightly clustered in the plot. Moreover, PCA analysis is showing that replicate samples have high variability in this phase (**Figure S2B**). Only after the second treatment, at BBCH61, and the third one at BBCH65, a more treatment-wise consistent clustering is observed.

The analysis of DEGs was set to compare samples across three treatment applications, two ANE doses, and two sampling time points (24h and 48h after treatment). The number of DEGs yielded by each comparison is shown in **Table 3**. Most of the genes were upregulated (62.5% of DEGs) after the first ANE application. Conversely, a greater number of down-regulated genes were identified after the second (70.5% of DEGs) and the third (57% of DEGs) applications.

**Table 3:** Number of differentially expressed genes with adj-p < 0.1 and |log_2_FC| ≥ 1, across treatment applications (1^st^, 2^nd^, and 3^rd^ applications), ANE doses (1 l ha^-1^ or 2 l ha^-1^), and sampling time (24h or 48h).

	1^st^ application		2^nd^ application		3^rd^ application	
	Up	Down	Up	Down	Up	Down
1 l ha^-1^ 24h *vs* NT 24h	–	–	67	133	38	67
1 l ha^-1^ 48h *vs* NT 48h	8	11	65	144	1	1
2 l ha^-1^ 24h *vs* NT 24h	12	1	12	78	17	20
2 l ha^-1^ 48h *vs* NT 48h	**-**	–	8	9	18	10

**Figure 4:**
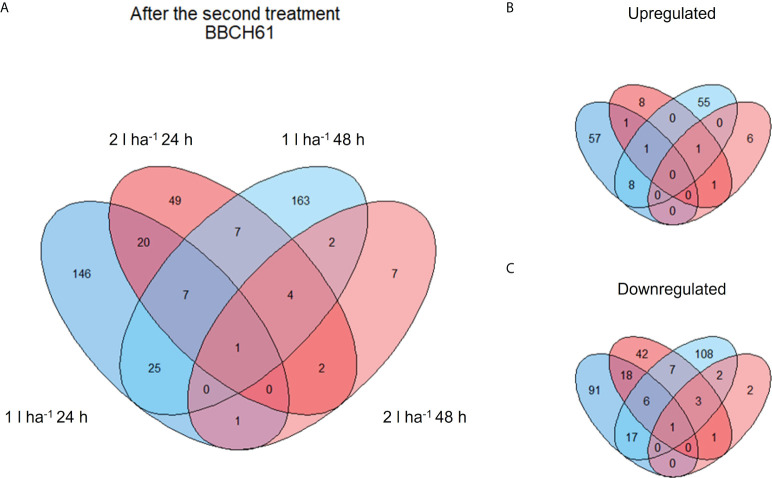
Venn diagram showing shared and unique DEGs of different comparisons after the second ANE application. The diagrams show the total number **(A)** and the breakdown between up- **(B)** and down- **(C)** regulated DEGs after the second ANE application at BBCH61.

**Figure 5:**
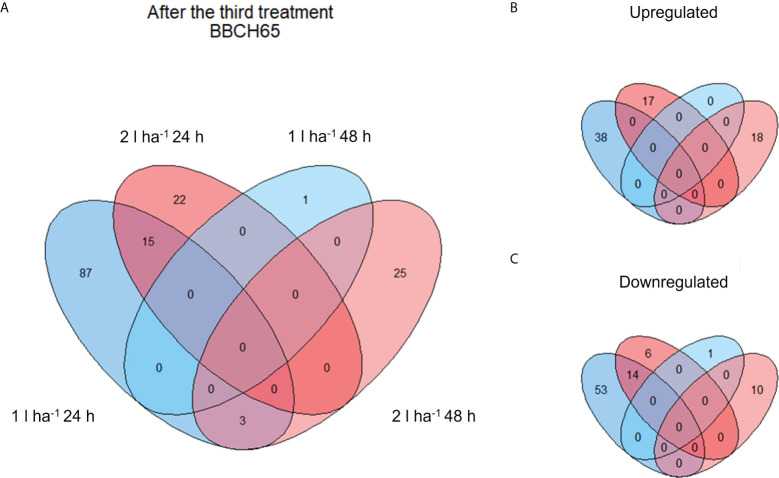
Venn diagram showing shared and unique DEGs of different comparisons after the third ANE application. The diagrams show the total number **(A)** and the breakdown between up- **(B)** and down- **(C)** regulated DEGs after the third ANE application at BBCH65.

A few genes were differentially expressed after the first treatment (**Table 3**), consistently with a non-ideal clustering of replicates observed in the PCA at the same stage. We assumed a weak biostimulant effect at BBCH51 and decided to focus on the results obtained from the second and third ANE applications, which yielded a higher number of DEGs and a more consistent PCA (**Table 3** and **Figure S2**). The number of DEGs shared between the two time points (24h and 48h) and two ANE doses within the same treatment application event, for both BBCH61 and BBCH65 were analysed (**Figures 4**, **5**). Only one gene, encoding a proline-rich protein 4-like, was consistently downregulated across all time points and ANE doses at BBCH61, whereas no gene was found to be mutually upregulated at both 24h and 48h and with both doses.

**Figure 6:**
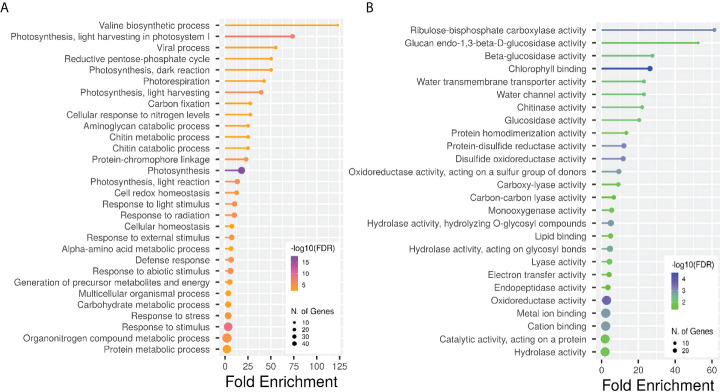
Gene ontology enrichment analysis for all the DEGs obtained across different comparisons. Lollipop plots show GO fold enrichment, significance (FDR ≤ 0.05), and number of genes in each pathway. GO categories analyses are biological process **(A)** and molecular function **(B)**. Analysis was perfomed with the online tool ShinyGO, v.0.66 (http://bioinformatics.sdstate.edu/go/).

**Figure 7:**
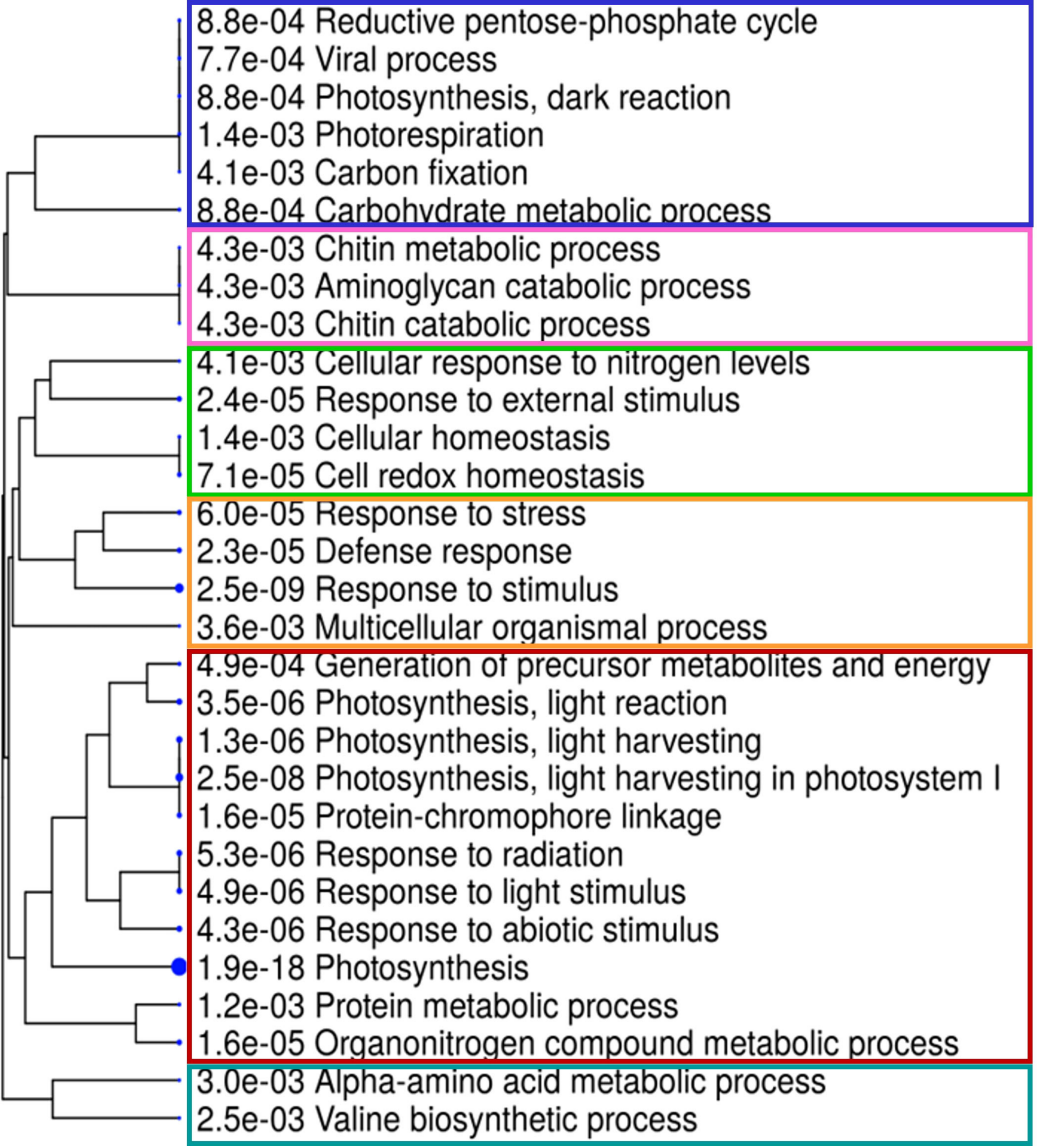
Hierarchical decision tree displaying the degree of association among enriched GO terms in the biological process and its statistical significance. Pathways with more shared genes are closer in the tree plot and visually grouped by different colors. Bigger dots indicate more significant p-values (ShinyGO, v.0.66, http://bioinformatics.sdstate.edu/go/).

A Gene Ontology (GO) enrichment analysis was conducted separately for DEGs obtained from different comparisons within each phenological stage and for every ANE dose and sampling time. The output for the most significantly enriched GO terms related to biological process and molecular function is presented in **Supplementary Table 4**. To better visualize and characterize the most relevant molecular mechanisms involved in the biostimulant activity, given the large number of different comparisons, we further conducted one GO enrichment analysis on the pool of the total number of DEGs obtained across all pairwise comparisons. The treatment mainly affected the expression of genes related to photosynthesis (both light and dark reactions), valine biosynthetic process, and response to several stimuli (**Figure 6**). The molecular functions GO terms with the greatest enrichment values were related to photosynthetic activity, among which are “ribulose-bisphosphate carboxylase activity”, “beta-glucosidase activity”, and “chlorophyll binding”. Interestingly, the GO terms “chitinase activity” and “water channel activity”, and those related to lipid binding and oxidoreductase and monooxygenase activity were also among the ones with greater fold enrichment values (**Figure 6B**). GO terms were hierarchically clustered based on shared genes. Such clustering produced six main groups (**Figure 7**) that show the main pathways affected by the ANE treatment. GO terms that are clustering together in the tree plots have more shared genes and larger dots indicate a lower p-value. This helps reduce the redundancy of GO terms and focus on the main broad categories enriched. They can be summarized in dark and light reactions in photosynthesis, chitin metabolic process, response to external stimulus, defense response, and biosynthesis of secondary metabolites. The broader categories and the ones with the highest significance in the decision tree are the categories of genes involved in photosynthesis and response to stimulus. To have an overview of the genes differentially expressed in each enriched broad category, a list of annotations and gene descriptions is provided in **Table 4**.

**Table 4:** A selection of representative genes differentially expressed in ANE-treated plants in at least one comparison.

Gene ID	Gene description
**Photosynthesis, dark reaction, and carbon fixation**	
Solyc02g085950	Ribulose bisphosphate carboxylase small subunit, chloroplastic 4
Solyc02g063150	Ribulose bisphosphate carboxylase small chain 1, chloroplastic
Solyc03g034220	Ribulose bisphosphate carboxylase small subunit, chloroplastic 2
**Chitin metabolic process**	
Solyc09g098540	Chitinase-like protein 1
Solyc10g055800	Endochitinase 4
Solyc10g055810	Chitinase
**Cell redox homeostasis**	
Solyc01g087850	Subtilisin-like protease
Solyc05g015490	Non-specific lipid transfer protein GPI-anchored 1
Solyc06g008760	Glutaredoxin-C13
Solyc10g007600	Glycolate oxidase
Solyc07g042440	Peroxiredoxin Q, chloroplastic
**Response to stimulus and stress**	
Solyc02g086820	Carbonic anhydrase
Solyc12g099970	SNF1 kinase complex anchoring protein
Solyc01g006300	Peroxidase
Solyc12g011450	Chlorophyll *a*/*b* binding protein 13, chloroplastic
Solyc01g006730	Calcium-dependent protein kinase 20-like
Solyc07g041720	Auxin-binding protein
Solyc05g055990	Aquaporin
Solyc10g048030	Kirola
**Photosynthesis**	
Solyc01g087040	Thylakoid lumenal 19 kDa protein, chloroplastic
Solyc01g102770	Photosystem II protein Z
Solyc02g069460	Photosystem I reaction center subunit III, chloroplastic
Solyc05g056050	Chlorophyll *a*/*b* binding protein 6A, chloroplastic
Solyc05g056070	Chlorophyll *a*/*b* binding protein precursor
Solyc10g075160	Ferredoxin
Solyc07g041720	Auxin-binding protein
Solyc02g064770	Probable esterase
Solyc04g073990	Annexin p34
Solyc01g087850	Subtilisin-like protease
**Biosynthesis of secondary metabolites**	
Solyc03g044330	Acetolactate synthase 2, chloroplastic
Solyc04g014510	Glutamine synthetase cytosolic isozyme 1-1
Solyc04g082030	Ornithine decarboxylase
Solyc08g007040	Glycine cleavage system H protein, mitochondrial

The categories are obtained through the clustering of GO biological processes most significantly enriched.

To validate the RNA-Seq data set, the expression levels of five candidate genes involved in photosynthesis and defense response selected among DEGs in at least two conditions, were measured by RT-qPCR on samples treated with ANE (2 l ha^-1^) after 24 h. Relative expression values of selected genes obtained with qPCR using the 2^-ΔΔCt^ method on plants treated with the 2 l ha^-1^ dose were compared with fold changes (FC) obtained from RNA-Seq analysis of plants treated with both ANE doses (**Table 5**). We observed some discrepancies between qRT-PCR and RNA-Seq data, particularly for *PIP1-7*, *KLUH/CYP78A5*, and *PR1b1*. We anyway observed an overall positive correlation between the relative expression values measured with qPCR and the FC obtained through sequencing. However, the use of biological replicates and the different normalization methods adopted may account for the differences observed in gene expression responses to the treatment. Moreover, the correlation was stronger for the RNA-Seq data obtained from samples treated with the lower dose of application (1 l ha^-1^) compared to the 2 l ha^-1^ dose which was the one used in the qPCR validation. The expression pattern of *RBSCs1*, *CA2*, and *KLUH/CYP78A5* detected by the RNA-Seq data after the second and third ANE applications (1 l ha^-1^) was generally consistent with the qPCR results (**Supplementary Figure 3**). However, the fold changes in up- and down-regulation of these genes in the treated samples compared to the control are not fully matching. Lower transcript levels of *RBSCs1* and *CA2*, encoding respectively a ribulose bisphosphate carboxylase small chain and a carbonic anhydrase, were observed in leaves of plants treated with 1 l ha^-1^ at both BBCH61 and BBCH65, whereas the only statistically significant down-regulation registered with higher ANE dose (2 l ha^-1^) is for *CA2* at BBCH61 (**Figure S3**). The *KLUH* gene, a member of the cytochrome P450 family, that controls fruit size and mass, modulates plant architecture, and ripening time (Chakrabarti et al., 2013), was upregulated in treated plants after the second application but was found downregulated in the same conditions at BBCH65. The *PR1b1* gene encoding a pathogenesis-related protein 1 was significantly upregulated after every treatment with the highest product dose (2 l ha^-1^) in the RNA-Seq results. The same higher level of the *PR1b1* gene transcript was observed in treated plants compared to untreated at BBCH65 in different biological replicates used for qPCR analysis, but not in the other two previous product applications in which we observed the downregulation of the same gene (**Figure S3**).

**Table 5:** RNA-Seq data validation of five candidate genes using RT-qPCR. Fold change in expression is presented using the 2^-ΔΔCt^ ± s.e. for RT-qPCR data and fold change for RNA-Seq data.

Gene ID	Gene name	Description	Treatment application	qRT-PCR	RNA-Seq	
				2 l ha^-1^	1 l ha^-1^	2 l ha^-1^
Solyc03g096290	*PIP1-7*	Aquaporin, plasmamembrane intrinsic protein 1.7	BBCH51	-1.88 ± 0.06	-1.01	1.74
			BBCH61	-3.24 ± 0.10	-4.11 *	-1.38
			BBCH65	2.34 ± 0.83	1.11	-9.13 *
Solyc03g114940	*KLUH/* *CYP78A5*	Cytochrome P450 78A5-like	BBCH51	1.01 ± 0.19	-1.51	-1.27
			BBCH61	1.04 ± 0.18	3.27 *	1.84
			BBCH65	-1.03 ± 0.03	1.22 *	1.23
Solyc02g063150	*RBSCs1*	Ribulose bisphosphate carboxylase small chain 1, chloroplastic	BBCH51	-1.05 ± 0.13	1.01	1.11
			BBCH61	-1.39 ± 0.20	-2.08 *	-2.39 *
			BBCH65	-1.05 ± 0.16	-5.55 *	-1.01
Solyc02g086820	*CA2*	Carbonic anhydrase	BBCH51	-1.11 ± 0.20	1.02	1.05
			BBCH61	-3.77 ± 0.06	-2.20 *	-2.66 *
			BBCH65	-1.35 ± 0.03	-7.09 *	1.13
Solyc09g007010	*PR1b1*	Pathogenesis-related leaf protein	BBCH51	-4.98 ± 0.16	2.95	132.52 *
			BBCH61	-3.19 ± 0.15	1.79	25.77 *
			BBCH65	4.65 ± 1.07	1.18	4.29 *

* Indicates genes significantly differentially expressed according to the adjusted p-value cutoff (p< 0.1).

### Effects of ANE treatment on tomato plants grown in greenhouse

**Figure 8:**
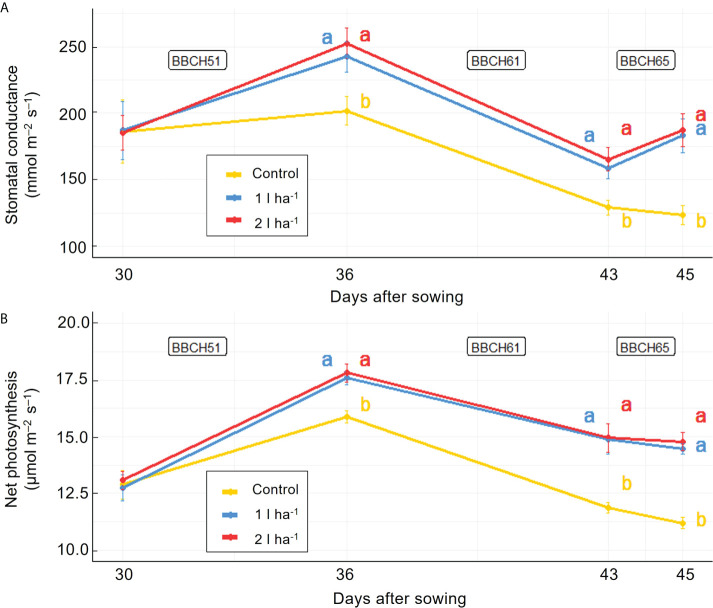
Effect of ANE treatment on photosynthetic parameters in tomato plants cultivated in greenhouse. Stomatal conductance **(A)** and net photosynthesis **(B)** were measured before the first treatment application and 48h after every ANE leaf application at BBCH51, BBCH61, and BBCH65 in Micro-Tom plants untreated (control) or treated with ANE (1 or 2 l ha^-1^). Each value is the mean of n = 6 observations ± s.e. Different letters indicate a significant difference according to LSD Fisher’s test (P ≤ 0.05).

**Figure 9:**
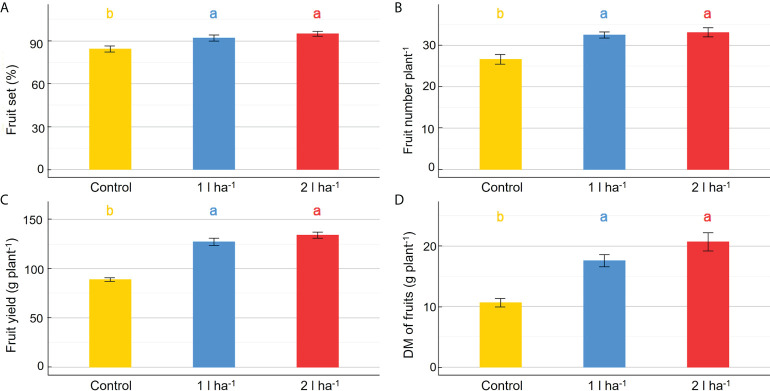
Effect of ANE treatment on fruit production in tomato plants cultivated in the greenhouse. The percentage fruit set **(A)**, the total number of mature fruits **(B)**, the total fruit yield **(C)**, and the total fruit dry biomass **(D)** were measured per plant in Micro-Tom cv untreated (control) or treated with ANE (1 or 2 l ha^-1^). Each value is the mean of n = 6 observations ± s.e. Different letters indicate a significant difference according to LSD Fisher’s test (P ≤ 0.05).

The physiological analysis carried out in the greenhouse showed significant effects of treatment on the average net photosynthesis and stomatal conductance, and a significant interaction between the biostimulant treatment and the time of application on net photosynthesis (**Table 6**). After the first application of ANE, the stomatal conductance and the net photosynthesis were both higher than the control at every time point, and the comparison between the two ANE doses revealed no statistically significant differences (**Figure 8**). Treated plants had an improved fruit set percentage, total fruit yield, and fruit dry biomass compared to untreated ones, and again no significant differences were found among the different ANE doses. (**Figure 9**).

**Table 6:** Mean values and analysis of variance of photosynthetic parameters after foliar application of ANE (biostimulant, B) at different phenological phases (time, T) in the greenhouse.

Treatment	Stomatal conductance (mmol m^-2^ s^-1^)	Net photosynthesis (µmol m^-2^ s^-1^)
**Biostimulant (B)**		
2 l ha^-1^	198 ± 8.7 a	15.1 ± 0.4 a
1 l ha^-1^	193 ± 9.3 a	14.9 ± 0.4 a
Control	160 ± 9.6 b	12.9 ± 0.4 b
**Time (T)**		
Before first treatment	186 ± 10.9 B	12.9 ± 0.3 C
BBCH51	232 ± 8.1 A	17.1 ± 0.3 A
BBCH61	151 ± 5.5 C	13.9 ± 0.5 BC
BBCH65	165 ± 9.3 BC	13.5 ± 0.4 BC
**ANOVA Significance**		
B	**	**
T	**	**
B x T	ns	**

Data are means ± standard error. Different letters indicate a significant difference according to LSD Fisher’s test (P ≤ 0.05). *, ** significant respectively at 0.05 or 0.01 levels according to ANOVA. BBCH51 (the first inflorescence visible: first bud erects), BBCH61 (first inflorescence: first flower open), BBCH65 (fifth inflorescence).

### Effects of ANE treatment on tomato plants grown in open field

Finally, the effects of ANE treatment were assessed in the open field. This step was to validate findings from the two previous experimental settings in controlled growth conditions. Regardless of the dose, the leaf gas-exchange parameters stomatal conductance and net photosynthesis were significantly (p ≤ 0.05) more important in treated plants at BBCH65 (**Table 7**). The leaf stomatal conductance and net photosynthesis of ANE-treated plants measured at the full flowering stage after the last ANE application were significantly (p ≤ 0.05) greater compared to non-treated plants. Before the beginning of the treatment, these leaf gas exchange parameters were similar among all groups of plants. Again, no difference was found between the two ANE doses.

**Table 7:** Mean values and analysis of variance of photosynthetic parameters after foliar application of ANE (biostimulant, B) at different phenological phases (time, T) in the field.

Treatment	Stomatal conductance (mmol m^-2^ s^-1^)	Net photosynthesis (µmol m^-2^ s^-1^)
**Biostimulant (B)**		
2 l ha^-1^	495 ± 13.9 a	25.5 ± 0.7 a
1 l ha^-1^	493 ± 16.5 a	25.3 ± 0.6 a
Control	428 ± 27.2 b	23.9 ± 1.0 b
**Time (T)**		
Pre-application	489 ± 8.6 A	26.3 ± 0.4 A
After last application	455 ± 23.6 B	23.5 ± 0.6 B
**ANOVA Significance**		
B	*	*
T	*	*
B x T	ns	ns

Data are means ± standard error. Different letters indicate a significant difference according to LSD Fisher’s test (P ≤ 0.05). *, ** significant respectively at 0.05 or 0.01 levels according to ANOVA.

Crop fruit yield and total biomass are important parameters in the open field. The total fruit yield and biomass of the total fruits were significantly affected by the biostimulant applications, but these variables did not differ between ANE doses (**Table 8**). The foliar application of biostimulant improved the yield of fresh tomato fruits by 35% (1 l ha^-1^), and 36% (2 l ha^-1^), in comparison with untreated plants, with no statistically significant difference between these two values.

**Table 8:** Analysis of variance of the yield and quality measured parameters that were affected by foliar application of the different ANE doses in the open field.

Treatments	Fruit yield (kg ha^-1^)	Fruit DM (kg ha^-1^)	Fruit set (%)
2 l ha^-1^	132750 ± 2612 a	5965 ± 170 a	95 ± 1.41 a
1 l ha^-1^	131650 ± 7800 a	5807 ± 474 a	96 ± 1.49 a
Control	97500 ± 6611 b	4294 ± 345 b	82 ± 2.93 b

Data are means ± standard error. Different letters indicate a significant difference according to LSD Fisher’s test (P ≤ 0.05)

## Discussion

The perspective of using plant biostimulants is hindered by the lack of knowledge translation from laboratory to field. A methodological framework is here presented, which includes different experimental settings in controlled and field conditions, to describe the effects of one biostimulant product through phenomics and transcriptomics. As a case study, an extract from the brown alga *Ascophyllum nodosum* was sprayed on tomato plants and applied at three time points during the flowering period.

The same experimental design was applied across three culture conditions. Firstly, a comprehensive picture of the plant responses induced by ANE, including physiological evaluations and global transcriptome analysis, was obtained in a climate chamber. Then, leaf gas exchange measurements and other yield-related morphological parameters were measured on plants grown in the greenhouse and field. Seaweed extracts can be applied as foliar spray or soil solutions. In this work, foliar applications were chosen with the aim of directly targeting the plant aerial organs at specific phenological stages.

The plant responses to the biostimulant treatment were conserved in the three different growing conditions, in terms of increased stomatal conductance, net photosynthesis, and key yield traits, such as the number of fruits and fruit biomass. Enhanced leaf stomatal conductance and rate of net photosynthesis were always detected after the third treatment application at full flowering. Also, in terms of regulation of gene expression, the response detected after the first ANE application was moderate compared to the one recorded after the second and third applications. These marked effects detected after the third ANE application suggest a cumulative effect of the treatments.

Overall, our observations were in line with previous studies showing increased tomato yields following the application of seaweed extracts (SWE) (Khan et al., 2009; Zodape et al., 2011; Ali et al., 2016; Murtic et al., 2018; Yao et al., 2020; Campobenedetto et al., 2021; Mzibra et al., 2021). This activity is partially explained by the reported presence in ANE of several hormones (e.g., auxins, cytokinins, gibberellins, abscisic acid (ABA), brassinosteroids, ethylene, and strigolactones) (De Saeger et al., 2020). Moreover, some ANE substances are known to stimulate the biosynthesis of endogenous phytohormones including auxins, cytokinins, and gibberellic acid, which leads to improved plant growth (Ali et al., 2019).

In many crops, including tomato, yield is associated with the number of flowers at maturity. Moreover, the cellular division phase leading to the fruit formation starts during flowering. Precisely, seaweed extracts were previously reported to promote flowering, increasing the number of flowers and fruits per cluster in tomato plants and yield parameters of other crops (Ali et al., 2016; Shukla et al., 2019; Hussain et al., 2021). Indeed, the product was applied during the flowering stage to evaluate possible effects on the fruit setting and the fruit yields eventually. The application of ANE improved fruit setting and yield across all experimental settings. Consistently, seaweed extracts modulated the expression of key genes involved in flowering (Dookie et al., 2021). Our results from the transcriptomic study point to “flower development” biological process at BBCH61 as a key functional category (**Table S4**). Indeed, *FLOWERING TIME* (*FT*), *CLAVATA* (*CLV*), and *SQUAMOSA PROMOTER BINDING-LIKE* (*SPL*) were up-regulated 24h after the lower ANE dose application. Salicylic acid (SA) has a widely reported flower-inducing activity and its accumulation can activate *FT* expression: in fact, SA-deficient plants show low levels of *FT* transcripts (Martínez et al., 2004). This suggests SA could be involved in flowering response to ANE.

In addition, greater fruit setting and yield in treated plants could be explained by greater photosynthesis and enhanced allocation of assimilates to the fruit. A possible explanation could be the increase in leaf chlorophyll content and photosynthetic capacity (Blunden and Gordon, 1986; Schiattone et al., 2018; Yao et al., 2020). Accordingly, Kumari et al. (2011) observed that the increase in vegetative growth could be due to an increase of photosynthetic pigments (chlorophyll and carotenoids) in the leaves of tomato plants treated with seaweed extracts. On the other hand, Xu and Leskovar (2015) described how the inhibition of gas exchange and stomatal conductance induced by drought stress on spinach, was reduced by *A. nodosum* extract but had no effect on leaf chlorophyll content, chlorophyll fluorescence, and gas exchange under full irrigation. The stomatal opening regulation and the photosynthesis modulation are primarily involved in the widely documented mitigation of drought stress detrimental effects exerted by seaweed extracts on plants (Santaniello et al., 2017; Shukla et al., 2018). When plants are grown in optimal conditions or in the field, without the environmental pressure of water stress, the effect of ANE treatment on the stomatal conductance was previously described either as an increased stomata opening (Salvi et al., 2019; Tombesi et al., 2021) or as an opposite reduced stomatal conductance (Santaniello et al., 2017). In the work by Santaniello et al. (2017), the decrease in the transpiration rate of ANE-treated *Arabidopsis thaliana* plants went with the reduced expression of the MYB60 transcription factor responsible for stomatal movements regulation, and a higher expression of two ABA-responsive genes, suggesting a priming effect on the plants that produced higher sensitivity of stomata to changes in ABA concentration.

The ANE used in the present work seemed not to target ABA-responsive genes. On the contrary, the stomatal conductance was promoted, and we observed the modulation of some SA-dependent genes. In the context of plant responses to biotic and environmental stresses, ABA is known to act antagonistically to SA, and to jasmonic acid and ethylene (Cao et al., 2011). Moreover, as previously reported, the recognition of ANE by the plant can induce the differential expression of defense-related genes compared to untreated control plants (Goñi et al., 2016; Omidbakhshfard et al., 2020) and among the genes dysregulated after the first ANE application in our study were some pathogenesis-related leaf proteins and a few endochitinases. The upregulation of some SA-dependent genes as *PR1b1* (Solyc09g007010), *FT* (flowering time, Solyc03g077920), and one *WRKY* transcription factor (Solyc03g095770) upon the ANE treatment encourages the hypothesis of the activation of the SA signaling pathway. Given the observed antagonistic interaction between SA and ABA, we hypothesize a diminished sensitivity to ABA that leads to reduced stomatal closure (Mosher et al., 2010).

The RNA-Seq results, when considering the pool of DEGs obtained from all the different comparisons, and the GO enrichment analysis output, are suggesting a substantial contribution of genes involved in several photosynthetic pathways. Both the biological processes of light-dependent reaction and the dark phase of photosynthesis are significantly enriched and mainly downregulated upon treatment application at BBCH61 and upregulated at BBCH65 (**Supplementary Table 4**). Overall, the transcriptome analysis revealed a major number of downregulated genes than upregulated ones. The same trend was recorded by Omidbakhshfard et al. (2020) 48 hours after spraying *Arabidopsis thaliana* plants with an ANE. Jannin et al. (2013) reported a greater number of downregulated compared to upregulated genes related to the photosynthetic pathways in shoots of *Brassica napus* after applying ANEs to the roots. In their work, the downregulation affected nuclear genes encoding chloroplast precursor proteins involved in biosynthesis and degradation of chlorophyll or a plastid division regulator. To the same group of chloroplast precursors, belonged upregulated genes (such as ferredoxins and carbonic anhydrase 1) encoding mainly proteins implicated in the electron transport chain.

Our results suggested an opposite regulation of two similar genes: a carbonic anhydrase gene (*CA2*) and a subunit of the Rubisco enzyme (*RBSCs1*). After one day from the leaf application of the ANE used in the present work, we recorded a downregulation of both genes in the early flowering stage and at full flowering (**Table 5**). At the same time, the physiological evaluation of the leaf gas exchange on the same plants was revealing a greater rate of stomatal conductance and net photosynthesis. The amount of CO_2_ that reaches the carboxylation sites can be modulated by the activity of beta carbonic anhydrases (CA*)*, which catalyzes the reversible hydration of CO_2_ to ${{\text{HCO}}_{3}}^{-}$ . The improved stomatal conductance of ANE-treated plants could account for optimal availability of CO_2_ reaching leaves carboxylation sites, thus resulting in a decreased *CA2* transcription. An overall increase in net photosynthesis rates was observed in treated plants as well as a downregulation of genes directly involved in the photosynthetic process. Thus, we can hypothesize that the untreated plants were undergoing photosynthetic apparatus early senescence. Possibly, coping with sub-optimal artificial light caused an increase in transcripts involved in the light reaction of photosynthesis.

Despite the physiological parameters measured and the yield traits never being influenced by the dose of the product, the lower dilution dose (1 l ha^-1^) seemed to induce a broader response in the plants in terms of the number of DEGs. Moreover, after the third application, the overall DEGs number decreased compared to the previous treatment. No gene was found to be up or downregulated by the treatment in more than two conditions (doses and sample timing). These patterns of gene expression modulation suggest either a dose-specific response to ANE, or an earlier common response that was not detected by sampling at 24h. Indeed, the DEG number decreased after 48h compared to 24h, especially for the greater ANE dose. Nevertheless, the final effect in terms of increased leaf gas exchange and fruit yield was achieved with both doses. Future applications of a similar methodology for biostimulant characterization could include more sample collection timings to achieve a more complete time-wise description of the molecular mechanisms involved in the plant response to the treatment.

## Conclusion

Across three growing conditions, tomato plants treated with ANE showed a greater number of flowers and fruit sets, resulting in a greater fruit yield. Also, net photosynthesis and stomatal conductance were improved after one ANE application. There was a transcriptome reprogramming caused by ANE treatment and particularly, after the second and the third leaf ANE application.

This study provides a detailed and robust methodology to evaluate plant biostimulant effects under different growing conditions. It also suggests that ANE application to tomato plants during flowering time can foster yield increases in greenhouse and field conditions. Furthermore, the combination of transcriptomic and phenomic approaches could become a key system for dissecting the plant response to any biostimulant. A comparison of morpho-physiological and molecular data collected under laboratory conditions showed coherent results. Such scientifically consistent methodological approaches to achieve the functional characterization of a biostimulant may support the whole stakeholders’ chain involved in developing, describing, registering, and commercializing plant biostimulants. Ultimately, farmers applying biostimulant products would greatly benefit from such a complementary study.

## Data availability statement

The datasets presented in this study can be found in online repositories. The names of the repository/repositories and accession number(s) can be found below: https://www.ebi.ac.uk/ena, PRJEB53962 (ERP138777).

## Author contributions

FM, AM, PS: conceptualization. FM, AM, and PS: supervision. FM, PS, AM, AB, ML, MB, GB, WZ-L, SR, CC, EP, and CH: methodology. AB, ML, CC, and PS: writing the original draft. FM, CC, SN, CH: writing, reviewing, and editing. All authors contributed to the article and approved the submitted version.

## Funding

This project was funded by Veneto Region in the framework of the PSR 2014–2020. Authors SR and MB were supported by Cariparo Foundation and PON Research &. Competitiveness MIUR - CUP C93H20000320007, respectively. CH is a research associate from F.R.S.-FNRS.

## Conflict of interest

Author Francesca Mangione is employed by Sipcam Italia S.p.A.

The remaining authors declare that the research was conducted in the absence of any commercial or financial relationships that could be construed as a potential conflict of interest. No funds from a commercial party were received in support of this article. No benefits in any form have been or will be received from a commercial party related directly or indirectly to the subject of this manuscript. No influence on the interpretation of the data and writing of the article was exerted by a commercial party.

## Publisher’s note

All claims expressed in this article are solely those of the authors and do not necessarily represent those of their affiliated organizations, or those of the publisher, the editors and the reviewers. Any product that may be evaluated in this article, or claim that may be made by its manufacturer, is not guaranteed or endorsed by the publisher.
