# Supplementary material for: A dual-omics approach for profiling plant responses to biostimulant applications under controlled and field conditions
Source: Front Plant Sci. 2022 Sep 26;13:983772. doi: 10.3389/fpls.2022.983772 (PMC9575556; doi:10.3389/fpls.2022.983772)
Supplement: Supplementary file 1 [file DataSheet_1.pdf]

Supplementary Material

Supplementary Figures and Tables

1.1 Supplementary Figures

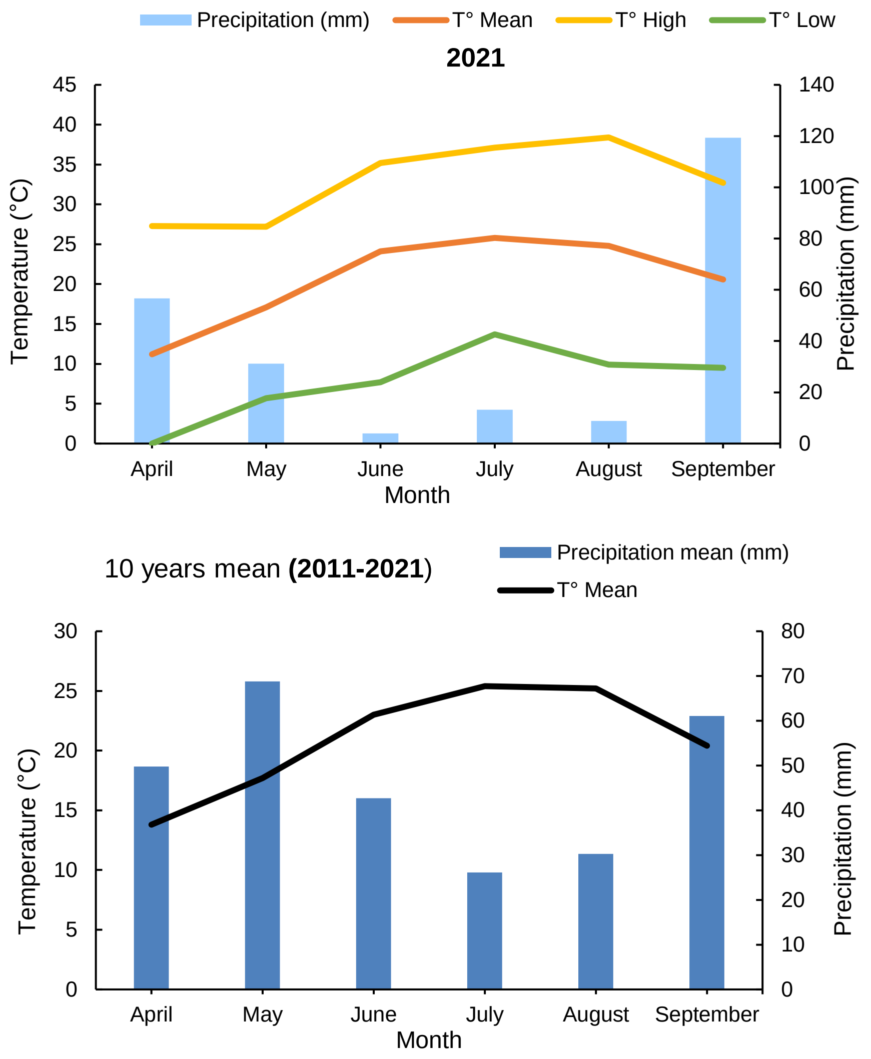

Supplementary Figure 1. Average monthly temperature, maximum and minimum temperatures, and rainfall precipitation during the experimental period in 2021 and in the previous ten years (2011-2021) at Cadriano, Bologna (Italy).

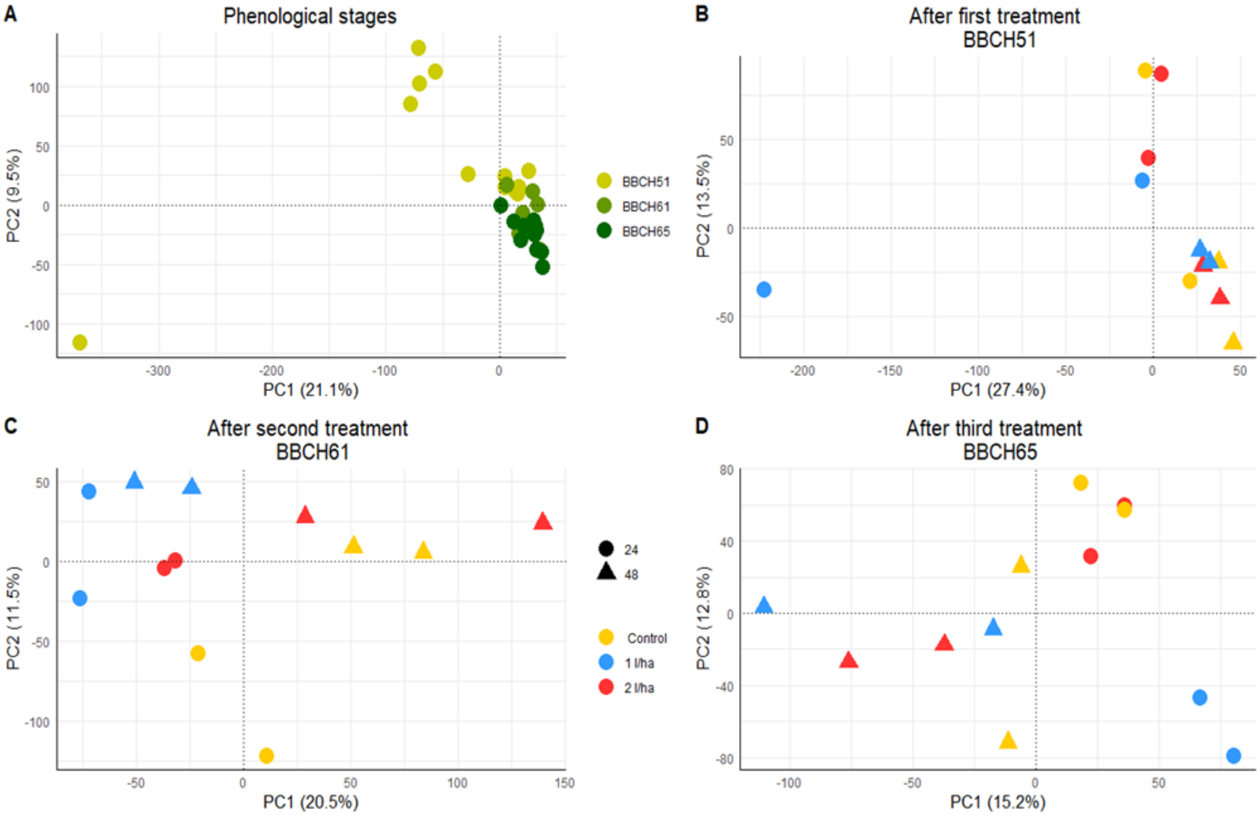

Supplementary Figure 2. Principal component analysis plots of log_2 normalized read counts in the three different treatment applications, at BBCH51, BBCH61, and BBCH65. Different colors refer to the treatment variable: blue and red are treated samples respectively with 1 and 2 l ha^{-1}. The untreated ones are yellow. Triangles and circles are used to distinguish sampling time, respectively samples collected 48 h and 24 h after the treatment.

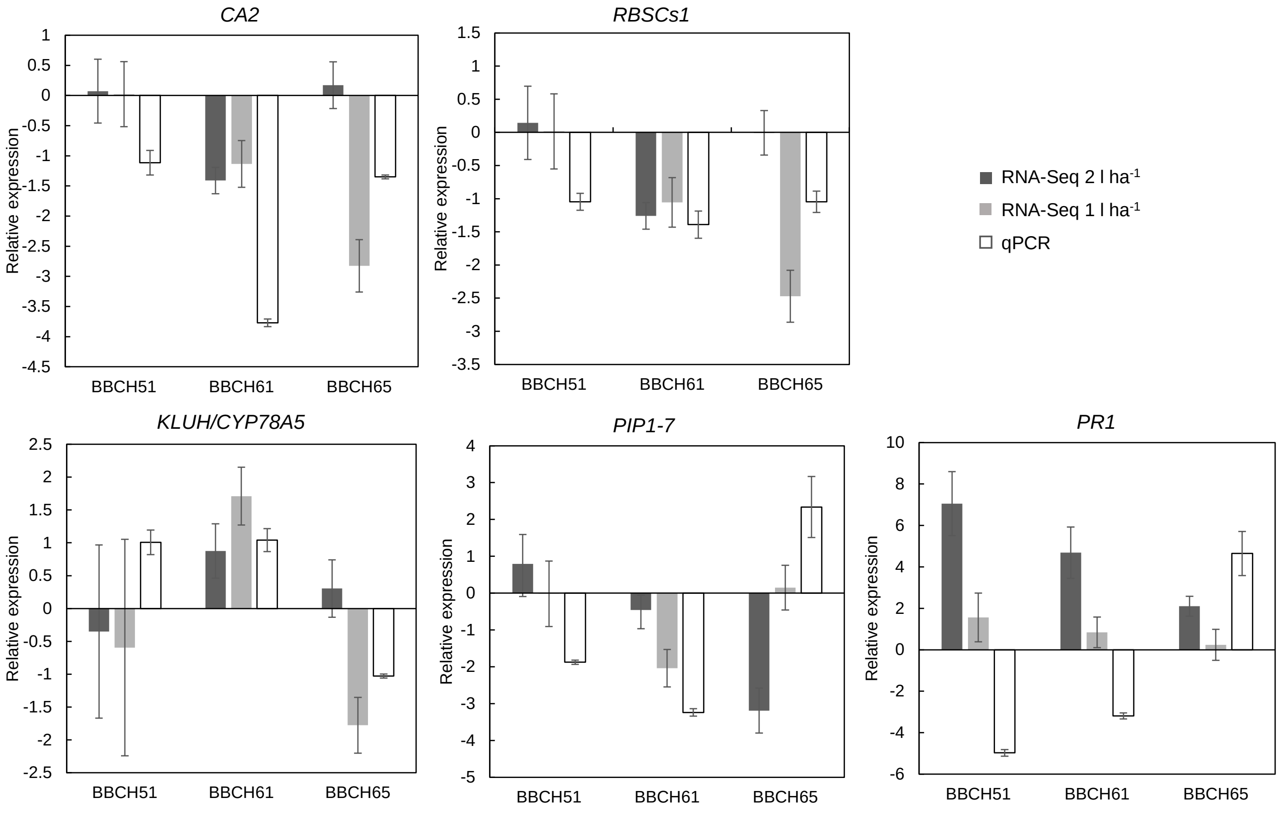

Supplementary Figure 3. Relative expression values ($2^{-\Delta\Delta Ct}$) from RT-qPCR of plants treated with 2l ha^{-1} dosage and log_2 FC from RNA-Seq for both doses of application of five genes in the three different times of treatment application (BBCH51, 61, and 65) after 24h.

1.2 Supplementary Tables

Supplementary Table 1. Physical and chemical properties of the substrate of cultivation.

Soil characteristics	Values
**Composition**	
White sod peat 10-25 mm (% vol.)	35%
White peat 0-25 mm (% vol.)	45%
Peat fiber (% vol.)	5%
Perlite (% vol.)	15%
**Structure**	
Medium (0-30 mm)	
**Chemical data**	
H value (H_2O, v/v 1:2:5)	6
Fertilizer level (g l^{-1})	1
Nitrogen (mg N l^{-1})	140
Phosphorous (mg P_2O_5 l^{-1})	160
Potassium (mg K_2O l^{-1})	180
Magnesium (mg mg l^{-1})	100
+ all necessary trace elements	
Iron added as EDTA chelates	
**Physical data**	
Dry matter	<10%
Water capacity	65-70%
Air capacity	20-25%

Supplementary Table 2. Selected genes used for validation of RNA-Seq data using real-time quantitative RT-PCR.

Gene ID	Gene name	Gene description	Forward Primer 5',3' and Reverse Primer 3',5'
Solyc03g114940.3.1	*P450*	Cytochrome P450 78A5-like	ACGCTGAAGTTGGAACCGAT<br>GCCTTGCCCACGAGAGTAAT
Solyc02g086820.2.1	*CA2*	Carbonic anhydrase	AGGGTGGATTTGAGCTGTGG<br>GAAGGAAATTGTGAGGGCCA
Solyc03g096290.3.1	*PIP1-7*	PIP1-7 aquaporin	TACAAAGAGCCACCACCAGC<br>TTAGAAACGCCCATGACGGT
			GCCGCTTCTTTTCCCGTTAC

Solyc02g063150.2.1	*RBCS-1*	Ribulose bisphosphate carboxylase small chain 1	CATGCATCTAACGCGTCCAC
Solyc09g007010.1.1	*PR1b1*	Pathogenesis-related leaf protein	TGACATATGAATCAAGTCAAACTCC<br>AATCAACTTAAGCCCATTATGAACA

Supplementary Table 3. Physical and chemical properties of the soil (0 - 30 cm depth).

**Soil characteristics**	**UM**	**Value**
Sand	% dm	24
Silt	% dm	48
Clay	% dm	28
pH		7.23
Total $CaCO_3$	%	1.02
Active $CaCO_3$	%	0.94
Organic C	g kg^{-1} dm	8.85
Organic matter	% dm	1.53
Total N	g kg^{-1}	1.16
P_2O_5	mg kg^{-1}	72
K_2O	mg kg^{-1}	170
C/N		7.63

Supplementary table 4. See Excel file.
